# Identification of Prognostic Model Based on Immune-Related LncRNAs in Stage I-III Non-Small Cell Lung Cancer

**DOI:** 10.3389/fonc.2021.706616

**Published:** 2021-10-20

**Authors:** Qiaxuan Li, Lintong Yao, Zenan Lin, Fasheng Li, Daipeng Xie, Congsen Li, Weijie Zhan, Weihuan Lin, Luyu Huang, Shaowei Wu, Haiyu Zhou

**Affiliations:** ^1^ Department of Thoracic Surgery, Guangdong Provincial People’s Hospital & Guangdong Academy of Medical Sciences, School of Medicine, South China University of Technology, Southern Medical University, Guangzhou, China; ^2^ College of Medicine, Shantou University, Shantou, China; ^3^ Guangdong Provincial People’s Hospital, Guangdong Academy of Medical Sciences, Guangzhou University of Chinese Medicine, Guangzhou, China; ^4^ The Second School of Clinical Medicine, Southern Medical University, Guangzhou, China; ^5^ Department of Thoracic Surgery, Jiangxi Cancer Hospital, Nanchang, China

**Keywords:** non-small cell lung cancer, long non-coding RNA, immune, prognostic model, tumor microenvironment

## Abstract

**Background:**

Long non-coding RNAs (lncRNAs) participate in the regulation of immune response and carcinogenesis, shaping tumor immune microenvironment, which could be utilized in the construction of prognostic signatures for non-small cell lung cancer (NSCLC) as supplements.

**Methods:**

Data of patients with stage I-III NSCLC was downloaded from online databases. The least absolute shrinkage and selection operator was used to construct a lncRNA-based prognostic model. Differences in tumor immune microenvironments and pathways were explored for high-risk and low-risk groups, stratified by the model. We explored the potential association between the model and immunotherapy by the tumor immune dysfunction and exclusion algorithm.

**Results:**

Our study extracted 15 immune-related lncRNAs to construct a prognostic model. Survival analysis suggested better survival probability in low-risk group in training and validation cohorts. The combination of tumor, node, and metastasis staging systems with immune-related lncRNA signatures presented higher prognostic efficacy than tumor, node, and metastasis staging systems. Single sample gene set enrichment analysis showed higher infiltration abundance in the low-risk group, including B cells (p<0.001), activated CD8+ T cells (p<0.01), CD4+ T cells (p<0.001), activated dendritic cells (p<0.01), and CD56+ Natural killer cells (p<0.01). Low-risk patients had significantly higher immune scores and estimated scores from the ESTIMATE algorithm. The predicted proportion of responders to immunotherapy was higher in the low-risk group. Critical pathways in the model were enriched in immune response and cytoskeleton.

**Conclusions:**

Our immune-related lncRNA model could describe the immune contexture of tumor microenvironments and facilitate clinical therapeutic strategies by improving the prognostic efficacy of traditional tumor staging systems.

## Introduction

Lung cancer has the leading rates of incidence and mortality worldwide, with the highest estimated deaths and 119,100 newly diagnosed cases in USA from Cancer statistic, 2021 (1). Non-small cell lung cancer (NSCLC) is the most common subtype, accounting for 85% of lung cancer cases. Target therapy and immune checkpoint inhibitors are emerging therapeutic strategies for NSCLC, but the identification of potential responders remains critical (2).

Long non-coding RNAs (lncRNAs) are diverse repertoires of RNA transcripts that are over 200 nucleotides in length, which lack the capacity for direct protein coding but are involved in the regulation of critical biological processes and cellular behavior (3). LncRNAs influence gene expression and have essential roles in carcinogenesis by combining regulatory molecules with proteins or directly binding to nucleic acids, protein complexes, or transcription factors (4). The roles of lncRNAs in immune systems are extensively investigated, and it has been summarized that lncRNAs are involved in the differentiation, activation, and function of immune cells. Emerging research has emphasized the role of lncRNAs in the regulation of carcinogenesis, immunosurveillance, and antitumor immune responses. (5) Specific lncRNAs have been found overexpressed in tumor-associated macrophages, shaping the tumor immune microenvironment and inhibiting tumor apoptosis (6).

Tumor infiltrating immune cells, compromising dendritic cells, mast cells, natural killer (NK) cells, macrophages, and tumor infiltrating lymphocytes (TILs), are present in the tumor microenvironment such as the tumor center peritumor stroma, or invasive margin (7). Immune checkpoints such as LAG-3, CTLA-4, and PD-L1 expressed in TILs are associated with cancer prognosis and therapeutic response, especially immunotherapy (8). It is evident that CD8+ T cells in tumor microenvironments are critical to the immune response. Recent studies have emphasized the function of tumor infiltrating B cells in immune surveillance and regulation of immunotherapy (9).

The traditional staging system for non-small cell lung cancer is the tumor, node, and metastasis (TNM) classification, which stratifies tumor subtypes and predicts cancer prognosis based on tumor size and its invasiveness to lymph nodes or distant organs, but does not take account of the tumor molecular and immune characteristics (5). Immune characteristics could be good candidates to improve prognostication of the TNM staging system in NSCLC (10).

Based on the fact lncRNAs could shape tumor microenvironments and predict the characteristics of NSCLC, we focused on immune-related lncRNAs to filter effective prognostic signatures. In this study, we extracted significant immune-related lncRNAs in stage I-III NSCLC and constructed immune-related lncRNAs based prognostic model, exploring the immune characteristics in tumor microenvironments and the potential therapeutic response of immunotherapy.

## Material and Methods

### Data Acquisition

All the clinical information and RNA-sequencing data of patients with NSCLC were downloaded from the Cancer Genome Atlas (TCGA) and the Gene Expression Omnibus (GEO). NSCLC patients with clinical stage I-III samples and complete follow-up information were included in this study. After sifting, 1357 cases with NSCLC from three data sets were incorporated into this study, including 970 patients from TCGA (https://portal.gdc.cancer.gov), 226 patients in GSE31210 (https://www.ncbi.nlm.nih.gov/geo/query/acc.cgi?acc=GSE31210), and 161 patients in GSE30219 (https://www.ncbi.nlm.nih.gov/geo/query/acc.cgi?acc=GSE30219). The 970 patients from TCGA were randomly separated in a 7:3 ratio to form the training cohort (n=717) and the testing cohort (n=253). GSE31210 and GSE30219 were combined into another independent validation cohort. Detailed baseline clinical features of three datasets are shown in **Table 1**. Batch effects were removed by the “ComBat” package of R software. Meanwhile, immune-related lncRNAs were downloaded and extracted from the Immlnc dataset (http://bio-bigdata.hrbmu.edu.cn/ImmLnc) (11).

**Table 1:** Characteristics baseline of patients in cohorts.

Variable	TCCA (N = 970)			GSE31210 (N = 226)	GSE30219 (N = 161)
	Training cohort (N = 717)	Testing cohort (N = 253)	*p* value		
Gender			0.1586		
Male	414 (57.7)	159 (62.8)		105 (46.5)	137 (85.1)
Female	303 (42.3)	94 (37.2)		121 (53.5)	24 (14.9)
Age	68.0 (33.0-99.0)	67.0 (40.0-85.0)	0.4507	61.0 (30.0-76.0)	62.0 (40.0-84.0)
AJCC pathologic stage			0.9050		
Stage I	383 (53.4)	140 (55.3)		168 (74.3)	135 (83.8)
Stage II	215 (30.0)	65 (25.7)		58 (25.7)	9 (5.6)
Stage III	119 (16.6)	48 (19.0)		0 (0.0)	17 (10.6)
AJCC T stage			0.8260		
T1	205 (28.6)	75 (29.6)			
T2	402 (56.0)	139 (54.9)			
T3	85 (11.9)	30 (11.9)			
T4	25 (3.5)	9 (3.6)			
AJCC N stage			0.924		
N0	463 (64.6)	165 (65.2)			
≥N1	254 (35.4)	88 (34.8)			
Survival status			0.4108		
Alive	428 (59.7)	159 (62.8)		188 (83.2)	101 (62.7)
Dead	289 (40.3)	94 (37.2)		38 (16.8)	60 (37.3)

### Construction and Validation of the Immune-Related LncRNA Model

The immune-related lncRNA model was identified using the training cohort, and the validation cohort and GEO datasets were used to validate the accuracy and efficacy of the model. We selected the immune-related lncRNAs by taking the intersection of lncRNAs between and TCGA and GEO datasets. The least absolute shrinkage and selection operator (LASSO) was chosen to reduce overfitting and to analyze the optimal immune-related lncRNA signature for predicting the overall survival of NSCLC patients. The “glmnet” R package was used for LASSO regression analysis. We calculated the risk score of each sample as follows: risk score = expression value of lncRNA 1 * coefficient + expression value of lncRNA 2 * coefficient + … + expression value of lncRNA n * coefficient. Then, the NSCLC patients were sorted into high-risk and low-risk groups based on the optimal cut-off value of the risk score. The area under the curve (AUC), performed by the “Survival” package of R software, was used to validate the sensitivity and specificity of the immune-related signature.

### Tumor Infiltrating Immune Cells Signature

Single sample gene set enrichment analysis (ssGSEA) was applied to quantify the immune infiltration level of 28 immune cell phenotypes. We obtained the gene set from previous studies, which included various immune cell phenotypes such as activated B cells, activated CD8 T cells, T follicular helper cell, and so on (12, 13). Also, CIBERSORT (https://cibersort.stanford.edu/) was applied to describe the abundance of 22 immune cell types in each NSCLC sample using R software. We evaluated the immune cell infiltration, stromal content, and tumor purity with the ESTIMATE algorithm. By comparing the results of ssGSEA, CIBERSORT, and ESTIMATE among high-risk and low-risk groups, we described the relationship between the tumor immune microenvironment and the immune-related lncRNA signature.

### Prediction of Potential Immunotherapy Response

A T cell dysfunction and exclusion signature was constructed by the tumor immune dysfunction and exclusion (TIDE, http://tide.dfci.harvard.edu/) algorithm to predict tumor immune escape, which could influence patients’ response to immunotherapy. We used TIDE score mapping to compare the potential clinical response to immune checkpoint inhibitors between high-risk and low-risk groups.

### Potential Biological Mechanisms of the Immune-Related LncRNA Model

We downloaded the single nucleotide variation (SNV) data from TCGA to analyze the difference of SNV among high-risk and low-risk groups through Fisher test. To explore the potential biological mechanisms, we used the “Limma” package of R software to identify the differentially expressed genes (DEGs) between high-risk and low-risk groups in training cohort. The gene ontology (GO) analysis and Kyoto Encyclopedia of Genes and Genomes (KEGG) pathway enrichment analysis were performed to analyze the functional enrichment of DEGs, using R software. The protein-protein interaction (PPI) network of DEGs was described by using the STRING database (Version 11.0) and constructed by using Cytoscape (Version 3.8.2). Then, gene set enrichment analysis (GSEA) was performed on GSEA software (http://software.broadinstitute.org/gsea/) by using the Molecular Signatures Database (MSigDB) Version 7.2 collections C2 (curated gene sets) (14).

### Verification of the LncRNA Expression Between NSCLC Tissues and Adjacent Normal Tissues by qRT-PCR

We collected sixteen paired NSCLC and adjacent normal tissue samples from Jiangxi Cancer Hospital after gaining ethical approval from the Human Research Ethics Committee in Jiangxi Cancer Hospital. Total RNA was isolated using RNAiso Plus (Takara) according to the manufacturer’s instructions. The RNA was reverse-transcribed using the Primer Script Reverse Transcriptase Reagent Kit with gDNA Eraser (Takara, RR047A). Real-time PCR was performed using the TB Green™ Premix Ex Taq™ (Takara, RR420A) and analyzed using the Bio-Rad CFX96 thermal cycler. The primer sequences used for the investigated genes are listed in online **Supplementary Table 3**. GADPH was used to standardize the gene expression. In order to compare the expression levels of lncRNA in different samples, the 2^-ΔΔCt^ method was adopted to calculate the expression levels of the immune-related lncRNA from the risk model.

### Statistical Analysis

Univariate cox proportional hazard regression was applied to assess the prognostic value of immune-lncRNA signatures by evaluating the association between risk score and overall survival in the training cohort. The correlation of the overall survival with immune-related lncRNA signature and the clinicopathological characteristics was calculated using the Kaplan-Meier curve. The Wilcoxon rank sum test and t-test were conducted for the comparison between two groups. Two-tailed P value < 0.05 was considered significant statistically. All statistical analyses were performed in R software, version 4.0.1.

## Results

### Construction and Verification of the Immune-Related LncRNA Model

**Figure 1:**
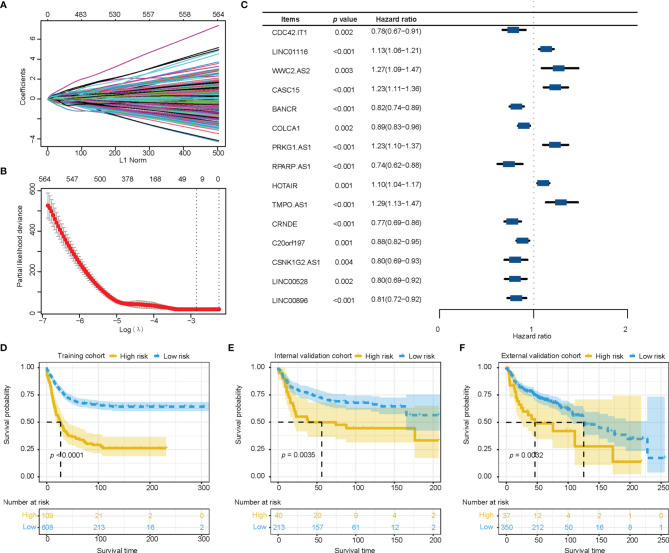
Construction and verification of the immune-related lncRNA prognostic model. **(A, B)** The least absolute shrinkage and selection operator was utilized to construct an immune-related lncRNA model. **(C)** The forest plot of fifteen immune-related lncRNAs was figured by multivariate cox regression analysis. **(D–F)** Survival analysis showed better survival among low-risk patients in training cohort, internal validation cohort, and external validation cohort. lncRNA, long non-coding RNA.

We extracted 1034 immune-related lncRNAs by taking the intersection among the lncRNAs in the TCGA-LUAD dataset, GEO and Immlnc database. We filtrated 15 immune-related lncRNAs with LASSO, then constructed the prognostic immune-related lncRNA model. (**Figures 1A, B**) Multivariate cox regression analysis showed potential prognostic properties in these 15 immune-related lncRNAs, and the expression level of 9 of the lncRNAs were positively associated with overall survival. Reciprocally, 6 immune-related lncRNAs were correlated with worse prognosis. All 15 lncRNAs are either significantly protective or risk factors for survival of stage I-III LUAD. (**Figure 1C**) Survival analysis of these 15 lncRNAs suggest significant survival differences between high and low expression levels of lncRNAs. (**Supplementary Figure 1**) Equation for the risk model from 15 significant immune-related lncRNAs is exhibited as follows:

Risk score = 0.0136*expression value of LINC01116 + 0.1285*expression value of WWC2.AS2 + 0.0415*expression value of CASC15 + 0.0545*expression value of PRKG1.AS1 + 0.1052*expression value of HOTAIR + 0.0011*expression value of TMPO.AS1 - 0.0711*expression value of CDC42.IT1 - 0.1914*expression value of BANCR - 0.0866*expression value of COLCA1 - 0.1751*expression value of RPARP.AS1 - 0.1610*expression value of CRNDE - 0.0422*expression value of C20orf197 - 0.1677*expression value of CSNK1G2.AS1 - 0.1954*expression value of LINC00528 - 0.0693*expression value of LINC00896.

Training cohort was divided into a high-risk group (n=109) and a low-risk group (n=608) according to the optimal cut-off value which was most significantly associated with overall survival. The high-risk group showed higher mortality than the low-risk group. The Kaplan-Meier survival analysis of the immune-related lncRNA signatures suggested better a survival probability in the low-risk group of the training cohort. (**Figure 1D**) Similar results for score distribution and survival analysis were found in the validation cohort and GEO cohort (**Figures 1E, F**).

### Clinical Value of Immune-Related LncRNA Model

Univariate cox regression analysis of the clinical characteristics and immune-related lncRNA model suggested that gender, AJCC T stage, AJCC N stage, AJCC TNM stage, and immune lncRNA model were significant prognostic factors for overall survival in NSCLC. All the significant candidates were included into multivariate cox regression analysis, indicating that AJCC T stage and immune-related lncRNA model were independent prognostic factors (**Table 2**).

**Table 2:** Univariate and multivariate analysis of clinical characteristics.

Variable	Univariate	p value	Multivariate	p value
	HR (95% CI)		HR (95% CI)	
Age	1.25 (0.98-1.58)	0.06	–	–
Gender	1.29 (1.04-1.59)	0.02	1.07 (0.86-1.32)	0.53
T stage		<0.001		
T1	1	–	1	–
T2	1.55 (1.20-2.00)	<0.001	1.31 (1.00-1.70)	0.047
T3	1.95 (1.38-2.74)	<0.001	1.63 (1.00-2.64)	0.048
T4	2.48 (1.51-4.09)	<0.001	1.79 (0.92-3.50)	0.088
N stage		<0.001		
N0	1	–	1	–
N1	1.54 (1.12-1.95)	<0.001	1.40 (0.92-2.11)	0.12
N2	1.91 (1.43-2.56)	<0.001	1.38 (0.69-2.78)	0.36
N3	0.87 (0.21-3.35)	0.85	0.79 (0.17-3.71)	0.77
NX	0.71 (0.23-2.23)	0.56	0.91 (0.29-2.85)	0.87
Tumor stage		<0.001		
Stage I	1	–	1	–
Stage II	1.44 (1.14-1.82)	<0.001	0.88 (0.57-1.34)	0.55
Stage III	2.04 (1.58-2.63)	<0.001	1.07 (0.50-2.30)	0.85
Purity	1.89 (0.98-3.63)	0.06	–	–
Risk score	2.40 (2.01-2.85)	<0.001	2.22 (1.85-2.66)	<0.001

**Figure 2:**
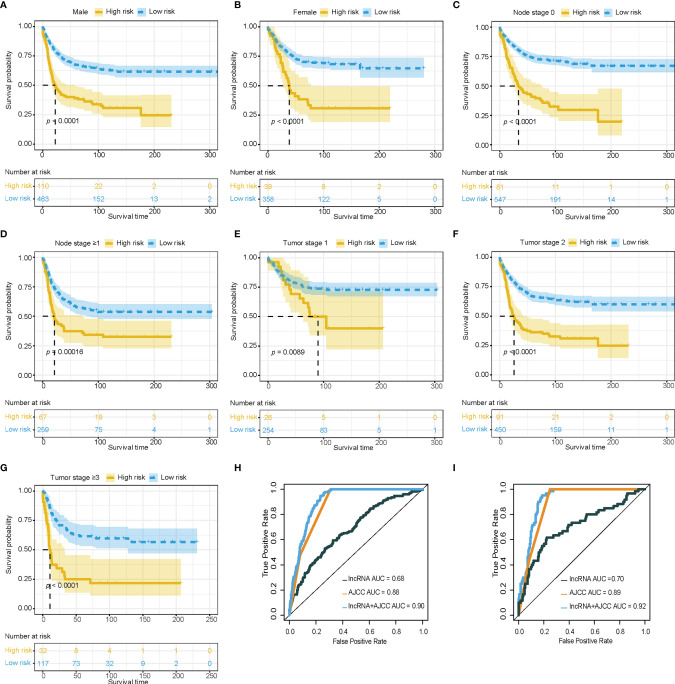
Clinical value of immune-related lncRNA model. **(A–G)** Survival analysis showed favorable survival for low-risk patients in different gender, node stage, and tumor stage. **(H, I)** Receiver operating characteristic curves were used to compare the predictive efficacy of the immune-related lncRNA based model alone, AJCC TNM staging alone, and the combination model in training cohort and validation cohort. lncRNA, Long non-coding RNA; AJCC, American Joint Committee on Cancer; TNM staging, Tumor, Node, and metastasis staging system.

To explore the robustness of the prognostic effect of the immune-related lncRNA model, Kaplan-Meier survival analysis was performed and stratified by clinicopathological characteristics, including T stage (T1, T2, T3-T4), N stage (N0, ≥N1), and gender (male, female). Similar results were found in the high-risk group, which had worse overall survival than low-risk group, and were delivered from different gender, T stage, and N stage in the training cohort (**Figures 2A–G**).

The area under the curve (AUC) of the combination between the AJCC TNM staging system and the immune-related lncRNA model was 0.90, while the AUC of AJCC TNM staging system alone was 0.88 in the training cohort for 3 years survival (**Figure 2H**). The combination of the AJCC TNM staging system and the immune-related lncRNA model presented a larger AUC than the AJCC TNM staging system alone in the internal validation cohort (AUC: 0.92 *vs* 0.89, **Figure 2I**) and external validation cohort (AUC: 0.64 *vs* 0.61, **Supplementary Figure 2**). Enhancement of prognostic accuracy of AJCC staging indicated a potential clinical application of the immune-related lncRNA model.

### Exploration of Immune Landscape and Immune Response

**Figure 3:**
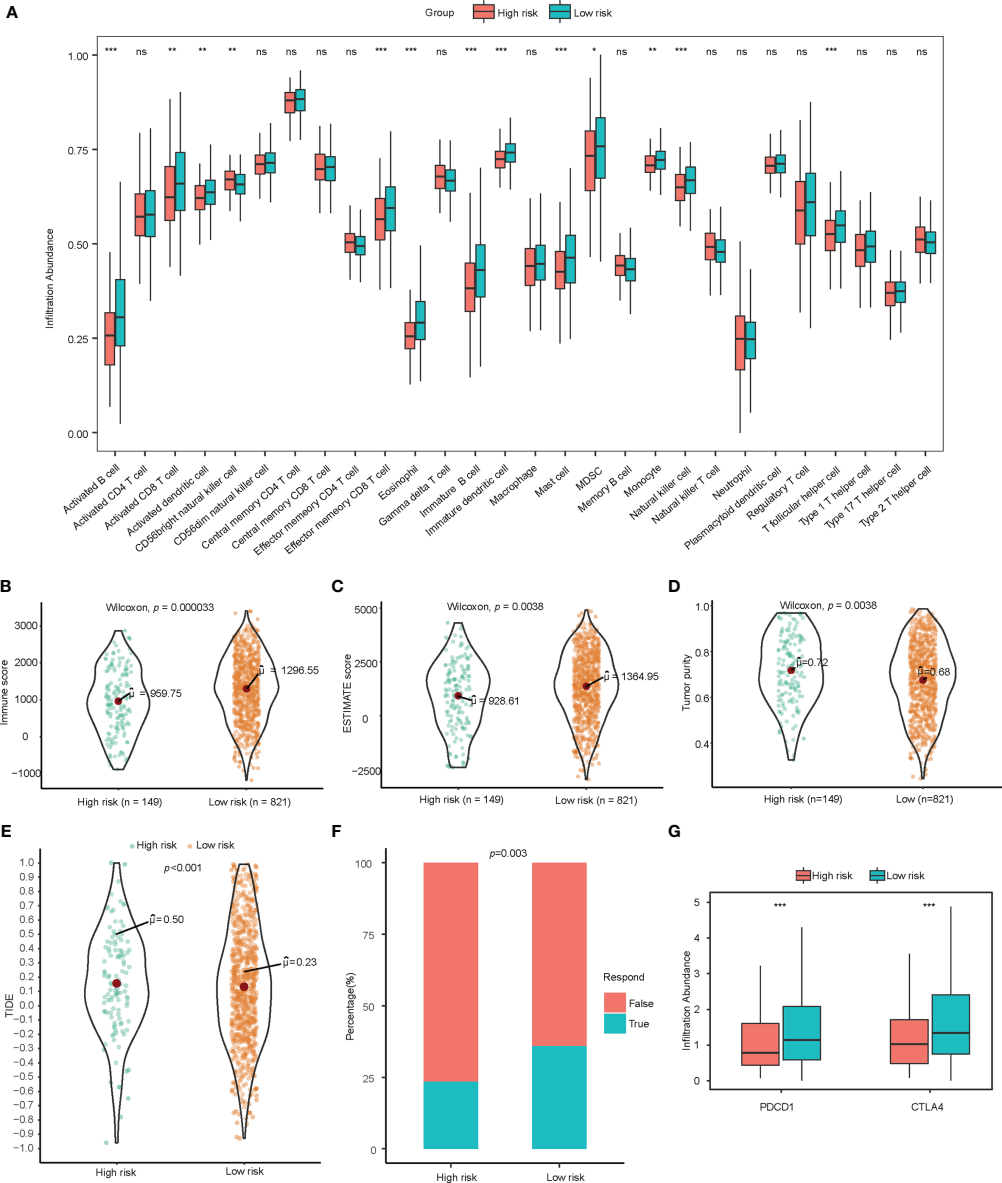
Exploration of immune landscape and immune response. **(A)** Single sample gene set enrichment analysis suggested a higher proportion of multiple immune cells such as activated B cells, CD8+ T cells, CD4+ T cells, and dendritic cells. ns for p>0.001, * for p<0.001, ** for p<0.0001, *** for p<0.00001. **(B–D)** Higher immune score, estimate score, and lower tumor purity are analyzed by ESTIMATE algorithm. **(E, F)** TIDE analysis estimated T cell dysfunction and exclusion and predicted response of immunotherapy. **(G)** Low-risk patients showed significantly higher PD-1 and CTLA-4. lncRNA, Long non-coding RNA; ssGSEA, Single Sample Gene Set Enrichment Analysis; TIDE, Tumor Immune Dysfunction and Exclusion. ns for p>0.05, * for p<0.05, ** for p<0.01, *** for p<0.001.

Tumor infiltrating lymphocytes analysis was conducted through ssGSEA and CIBERSORT. Through ssGSEA, almost all of the infiltrating immune cells showed higher infiltration abundance in low-risk group, especially activated B cells (p<0.001), immature B cells (p<0.001), effector memory CD4 T cells (p<0.001), activated CD8 T cells (p<0.01), effector memory CD8 T cells (p<0.001), activated dendritic cells (p<0.01), immature dendritic cells (p<0.001), CD56+ Natural killer cells (p<0.01), mast cells (p<0.001), monocytes (p<0.01), and T follicular helper cells (p<0.001) (**Figure 3A**). A higher proportion of CD8+ tumor infiltrating lymphocytes could be detected in the low-score group with CIBERSORT (**Supplementary Figure 3**). Tumor purity as calculated by the ESTIMATE algorithm was not a significant prognostic factor for NSCLC based on univariate cox regression analysis in our study (**Table 2**).

To assess the immune infiltration in histological aspects, the ESTIMATE algorithm was used to infer the proportion of stromal cells and immune cells in tumor tissue. The low-risk group has higher immune score (p<0.001) and estimate score (p=0.004) significantly, which indicates more immune cell infiltration and lower tumor purity (p=0.004). (**Figures 3B–D**) ESTIMATE analysis verified the results of the tumor infiltrating lymphocyte evaluation with ssGSEA and CIBERSORT.

Observing the correlation between the immune-related lncRNA model and the tumor microenvironment, we further explored the association between this model and the response of immunotherapy. The TIDE score integrates the T cell dysfunction and exclusion signature to evaluate tumor immune escape. The high-risk group calculated from immune-related lncRNA signature was found to have a higher TIDE score, indicating potential tumor T cell dysfunction and exclusion. The predicted proportion of responders to immune checkpoint blockade was lower in the high-risk group as well. (**Figures 3E, F**) In addition, among patients in low-risk group, the gene expression of *PDCD1* and *CTLA-4* were significantly higher than those in high-risk group (**Figure 3G**).

### Somatic Mutation and Pathway Analysis of the Immune-Related LncRNA Model

In order to compare the difference of somatic mutation of the immune-related lncRNA model, we used the “maftools” R package to calculate the SNV among the high-risk and low-risk groups. We found that the high-risk group had high mutation of TP53, TTN, MUC16, RYR2, CSMD3, XIRP3, USH2A, ZFHX4, LRP1B, and KEAP1 (**Supplementary Figure 4A**). The low-risk group was characterized by frequent mutation of TP53, TTN, MUC16, CSMD3, RYR2, LRP1B, USH2A, ZFHX4, KRAS, and FLG (**Supplementary Figure 4B**). Meanwhile, we analyzed the level of EGFR mutation in the two groups and we found no difference between the high-risk group and low-risk group (Fisher test, p=0.246, **Supplementary Figure 4C**).

**Figure 4:**
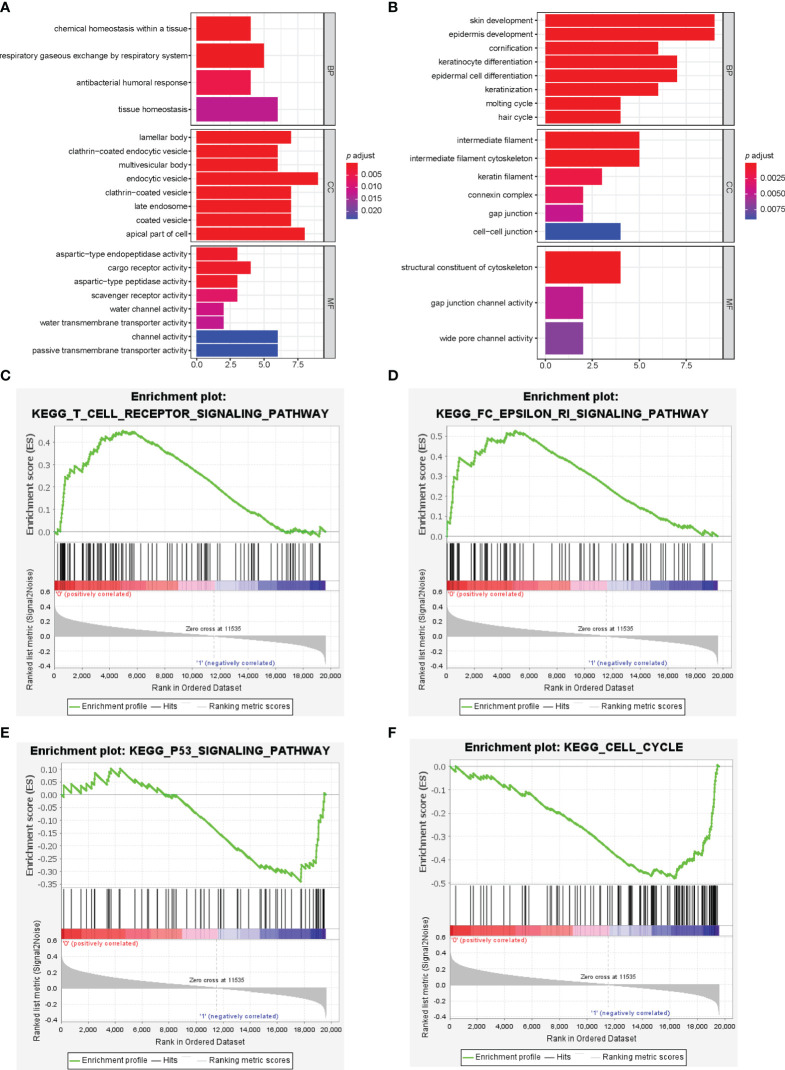
Pathway analysis of immune-related lncRNA model. **(A, B)** Gene ontology analysis was used to explore the potential functional mechanism of immune-related lncRNA model, and the results are visualized in the low-risk group **(A)** and high-risk group **(B)**. Immune-related lncRNA signaling pathway obtained by gene set enrichment analysis, including T cell receptor signaling pathway **(C)**, Fc epsilon RI pathway **(D)**, p53 signaling pathway **(E)**, and cell cycle **(F)**.

We identified 19592 differentially expressed genes (DEGs) between the high-risk and low-risk groups, among which 72 DEGs were significant (p<0.05) and had fold change ≥2. GO functional enrichment analysis showed up-regulated DEGs for the low-risk group enriched in humoral immune response and channel activity, and down-regulated DEGs for the low-risk group enriched in skin development, keratinocyte differentiation, and cytoskeleton. (**Figures 4A, B**) KEGG pathway analysis indicated significant enrichment in the PI3K-AKT signaling pathway, Ras/Rap signaling pathway, MAPK signaling pathway, and regulation of actin cytoskeleton. (**Supplementary Figure 5**) GSEA showed significantly positive enrichment of the Fc epsilon RI pathway, asthma, vascular smooth muscle contraction, T cell receptor signaling, B cell receptor signaling, renin-angiotensin-aldosterone system, and VEGF signaling pathways in the low-risk group. (**Figures 4C, D** and **Supplementary Table 1**) Meanwhile, up-regulated pathways enriched in high-risk group were DNA replication, cell cycle, nucleotide excision repair, p53 signaling, homologous recombination, mismatch repair, pentose phosphate pathway, and tricarboxylic acid cycle. (**Figures 4E, F**; **Supplementary Figure 6** **and** **Supplementary Table 2**).

### Expression Level of 15 Immune-Related LncRNAs Between NSCLC Tissues and Adjacent Normal Tissues by qRT-PCR

Finally, we measured the expression levels of 15 immune-related lncRNA from the risk model in the sixteen paired NSCLC and adjacent normal tissues using qRT-PCR. The result showed that three lncRNAs (CASC15, BANCR, and RPARP.AS1) had low expression in NSCLC tissues when compared with normal tissues (**Supplementary Figure 7**).

## Discussions

We constructed a 15 immune-related lncRNA based model using the LASSO algorithm for NSCLC in the TCGA dataset and validated it using the GEO datasets. Combined model demonstrated an enhanced prognostic efficacy compared to AJCC TNM staging system alone. A higher proportion of immune cell infiltration was detected in the survival-benefit group stratified by our model. The immune infiltration score described by the ESTIMATE algorithm is higher in the survival-benefit group, which is predicted with better immunotherapy response reasonably. GO functional analysis showed an enrichment in the humoral immune response, while KEGG showed enrichments in the PI3K-AKT signaling pathway, MAPK signaling pathway, and Ras/Rap signaling pathway. GSEA showed enrichments in the Fc epsilon RI pathway, asthma, T cell receptor signaling, and B cell receptor signaling.

Our study constructed and validated an immune-related lncRNA signature for stage I-III NSCLC with good prognostic AUC in all enrolled datasets. A total of 15 lncRNAs were identified to construct the immune-related lncRNA model, including 9 lncRNAs (CDC42.IT1, BANCR, COLCA1, RPARP.AS1, CRNDE, C20orf197, CSNK1G2.AS1, LINC00528, and LINC00896) associated with better overall survival and 6 lncRNAs (LINC01116, WWC2.AS2, CASC15, PRKG1.AS1, HOTAIR, and TMPO.AS1) correlated with worse prognosis. Some of these lncRNAs had been confirmed to be related to cancer progression and prognosis in previous studies. CASC15 has been reported to be upregulated in various types of tumor tissues (15), including NSCLC. As part of HIF-1α/CASC15/SOX4/β-catenin axis, CASC15 plays an essential role in cell proliferation, invasion, and tumor development in NSCLC (16). Meanwhile, CASC15 can promote lung cancer metastasis *via* miR-766-5p/KLK12 axis (17). *Homebox* (HOX) are vital in embryonic development and oncogenesis and the most studied HOX-lncRNAs is HOTAIR. HOTAIR is also significantly upregulated in NSCLC and is known for its association with higher TMN staging, lymphatic metastasis, and poor prognosis (18). The previous studies had found that HOTAIR could promote the level of miR-149-5p to facilitate the process of invasion, migration, and cell proliferation in NSCLC (19). Furthermore, HOTAIR has been linked to drug resistance in several types of tumor. Silencing HOTAIR expression can revert the gefitinib resistance of lung adenocarcinoma (20). Several studies have emphasized the potential value of LINC01116 as a prognostic marker or therapeutic target in various kinds of cancer. LINC01116 has been confirmed to accelerate tumor progression by regulating tumor-associated genes such as *MYC (* 21) and *p53 (* 22). In lung cancer, the upregulation of LINC01116 is an important reason for tumor cell proliferation and migration as it enhances the process of epithelial-mesenchymal transition (EMT) (23). In addition, as one of effectors in the IFN/STAT1 pathway, IFI44 is repressed by LINC01116, leading to acquired resistance of gefitinib in NSCLC (24). TMPO.AS1 performs its tumor-promoting function *via* activating the PI3K/AKT/mTOR pathway in gastric cancer and HCC (25). However, studies on BANCR and CRNDE function on tumor procession have shown conflicting results. For BANCR, some researchers have identified its influence in tumor invasion and migration (26). In contrast, overexpression of BANCR can control the content of N-cadherin, and vimentin and E-cadherin were shown to inhibit EMT in another study. (27) In our study, BANCR was used as a positive prognostic predictor for overall survival in NSCLC. As for CRNDE, most studies show that CRNDE can promote cell proliferation, invasion, and migration and inhibit apoptosis in colorectal cancer, lung cancer, glioma, and other cancers. CRNDE was also shown to affect cancer microenvironments and metabolism *via* the PI3K/AKT/mTOR and Raf/MAPK pathways. In conclusion, CRNDE might be a potential cancer promoter (28). However, recent research indicates the unique function of CRNDE in attenuating chemoresistance in gastric cancer by reducing the stability of *SRSF6* (29). Meanwhile, our immune-related lncRNA signature also describes CRNDE as a favorable factor for overall survival in NSCLC. KEGG pathway analysis of our signature also shows enrichment of the PI3K/AKT and MAPK signaling pathways. The molecular mechanism of CRNED in cancer prognosis has not been completely investigated, and requires additional experiments to explore it in the future.

The TNM staging system is a cancer staging manual constructed by the American Joint Committee on Cancer on anatomic extent, in the pursuit of definitive prognoses and selecting the most beneficial therapeutic strategies. However, clinical outcomes vary among different patients in the same stage due to diverse biological behavior determined by molecular and genetic features (30). Immune contexture represents the results of dynamic interaction between tumor cells and the immune system in the tumor microenvironment. Prognostic immune parameters have been studied to predict the prognosis of cancer. Immunoscore is an unprecedented biomarker describing the proportion of immune cells in the tumor centre, invasive edge, and peritumor stroma (31). In colorectal cancer, immunoscore is used to predict prognosis, therapeutic effects, and disease relapse after immune checkpoint inhibitor therapy (32). With respect to NSCLC, a Norwegian study identified stromal CD8+ density and CD45RO+ memory T lymphocytes as independent prognostic factors for NSCLC regardless of endpoints, and proposed them as a supplement to the TNM-staging system (10). Prospective multicenter clinical trials are designed to evoke the attention of TNM-immunoscore for clinical application (33). After identifying prognostic immune-related lncRNA signatures, the differences of immune contexture including tumor infiltrating lymphocytes and immunescore were explored with our immune-related lncRNA model. An improvement of prognostic efficacy was found in the training cohort, internal validation cohort, and external validation cohort, and provides a novel immune indicator as a nonanatomic supplement of tumor features for the TNM staging system.

Infiltration of different types of immune cells is associated with cancer progression and patient survival in NSCLC (34). Our study identified differences in tumor infiltrating cells between high-risk and low-risk groups according to an immune-related lncRNA model, focusing on cells including dendritic cells, B cells, CD4 T cells, CD8 T cells, Natural killer cells, T follicular helper cells, and mast cells. Cytotoxic CD8+ T lymphocytes (CTLs) are essential immune cells against tumor cells (35). The priming of CTLs requires antigen presentation and co-interaction with mature dendritic cells, natural killer cells, and CD4+ T cells. Mature dendritic cells and natural killer cells, motivated and licensed by CD4+ T cells, secret costimulatory molecules and cytokines, and then CTL is priming (36). Infiltrating B cells are emphasized as active participants that orchestrate the antitumor immune response. B cells are involved in antigen presentation to T cells directly, or facilitate the antigen uptake of dendritic cells (37). T follicular helper cells rely on antigen-specific B cells, and reciprocally facilitate B cell proliferation and differentiation, which is crucial in humoral response (38). Significant associations have been verified by multiple studies between the clinical outcome of cancer patients and CD4+ T cells, CD8+ T cells, B cells, dendritic cells, natural killer cells, and mast cells (39, 40).

In the present study, we inferred that atopy may be relevant to the development and prognosis of lung cancer based on evidence that the low-risk group had a higher infiltration of mast cells, and enriched Fc epsilon RI pathways, asthma and vascular smooth muscle contractions from GSEA. An up-to-30 year prospective study with 37747 participants in Denmark observed a 10-fold higher IgE level in non-Hodgkin lymphoma, oral or pharynx cancer, lung cancer, and esophagus cancer. However, these results were non-significant after multivariable adjustment (41). Recent research based on the Surveillance, Epidemiology, and End Results (SEER) database in the United States suggested asthma was associated with reduced risk of liver cancer, which could be attributed to the activation of immunosurveillance from allergic response (42). The cellular mechanism of the antitumor function of IgE/FcϵRI combination could be explained by cross-presentation with dendritic cells to induce the priming of cytotoxic T lymphocytes (43). High-affinity IgE receptors and Fc epsilon RI signaling pathways constituted the most significant pathway of prognostic signature for lung adenocarcinoma. MS4A2, an IgE receptor related gene expressed in tumor-infiltrating mast cells, was an independent favorable prognostic biomarker (44). Ultra-low IgE is correlated with a higher risk of malignancy and could be a diagnostic and prognostic biomarker for lung adenocarcinoma (42). Hence, our results validate the critical role of IgE and allergic reactions in the antitumor response of low-risk patients, stratified based on immune-related lncRNA signature.

Immune checkpoint inhibitor (ICI) immunotherapy has emerged as an effective treatment for various kinds of cancers, including NSCLC (45). However, only 20%-30% patients with NSCLC respond to immunotherapy. Predictive biomarkers, such as PD-1 (programmed cell death protein 1) and CTLA-4 (cytotoxic T lymphocyte-associated antigen-4), are frequently used to assess the response of ICI in NSCLC. In our study, immune checkpoint genes such as PD-1 and CTLA-4 were significantly higher in the low-risk group than in high-risk group (46). The TIDE scores were also significantly higher in the low-risk group. Thus, patients in the low-risk group may have better response to ICI therapy. Tumor-infiltrating immune cells have also been regarded as a predictor for response to immunotherapy (46). For example, KEYNOTE-001 has found that a high percentage of CD8+ T cell infiltration showed a strong association with superior ICI treatment responses when treated with pembrolizumab. (47) We also found that the low-risk group showed higher immune cells infiltration abundance, such as activated B cells, immature B cells, CD8+ T cells, and so on. In conclusion, our 15-immune-related lncRNA signature was closely related to ICI response.

Some limitations of this study should be considered when interpreting the results. First, all of the results were completed based on retrospective studies and public datasets. The accuracy of our immune-related lncRNA signature should be further verified with a clinical real-world dataset. Second, laboratory explorations are needed to verify and illuminate the molecular mechanisms of these immune-related lncRNAs. Finally, as the predictive marker of ICI response, TIDE score is just verified in several datasets. Hence, available immunotherapy cohorts are warranted to confirm the clinical application of the immune-related lncRNA signature.

In conclusion, we established a 15-immune-related lncRNA prognostic model for NSCLC. This model could be applied in clinical situations as a supplement to the TNM staging system for an improvement in the predictive efficacy of cancer malignancy and prognosis. Low-risk patients stratified by our model have higher infiltration of immune cells such as dendritic cells, CD8+ T cells, CD4+ T cells, B cells, natural killer cells, and mast cells. Pathway analysis of our immune-related lncRNA signature might indicate an underlying mechanism associated with humoral immunity, cell-mediated immunity, and the regulation of the cell cycle.

## Data Availability Statement

The original contributions presented in the study are included in the article/**Supplementary Material**. Further inquiries can be directed to the corresponding author.

## Author Contributions

QL, LY, and HZ designed this work. ZL performed experiments such as qRT-PCR. FL and DX integrated the data and conducted the analyses. CL and WZ wrote this manuscript. WL, LH, and SW edited and revised themanuscript. All authors contributed to the article and approved the submitted version.

## Funding

Guangzhou Municipal Science and Technology Project and Natural Science Foundation of Guangdong Province.

## Conflict of Interest

The authors declare that the research was conducted in the absence of any commercial or financial relationships that could be construed as a potential conflict of interest.

## Publisher’s Note

All claims expressed in this article are solely those of the authors and do not necessarily represent those of their affiliated organizations, or those of the publisher, the editors and the reviewers. Any product that may be evaluated in this article, or claim that may be made by its manufacturer, is not guaranteed or endorsed by the publisher.
