# Supplementary figures and images for: Identification of Prognostic Model Based on Immune-Related LncRNAs in Stage I-III Non-Small Cell Lung Cancer
Source: Front Oncol. 2021 Oct 20;11:706616. doi: 10.3389/fonc.2021.706616 (PMC8564147; doi:10.3389/fonc.2021.706616)

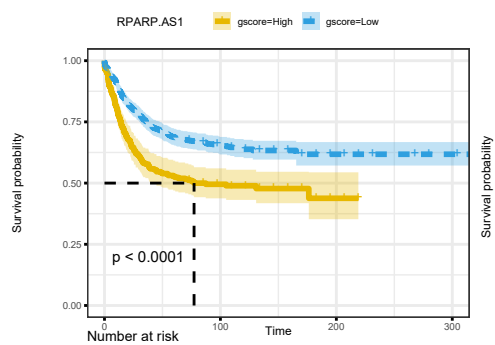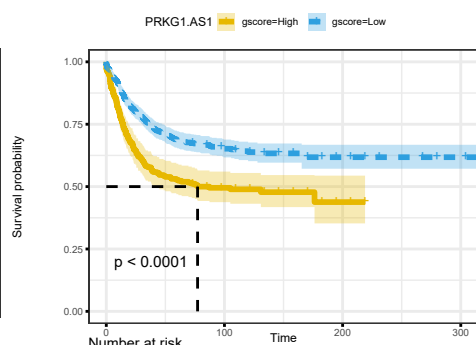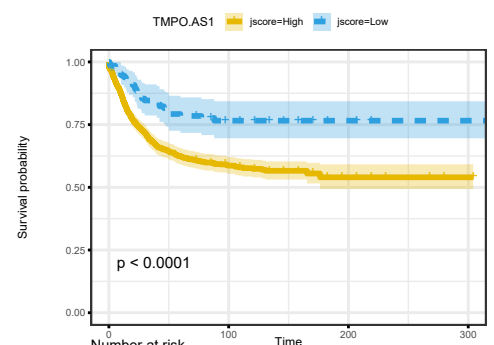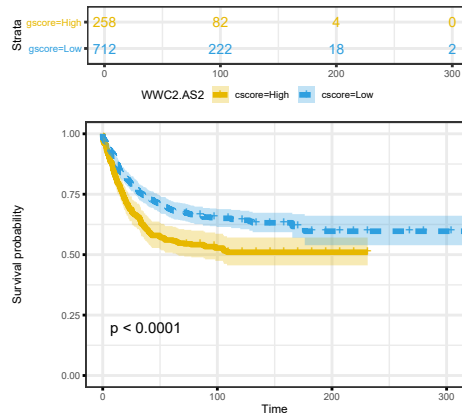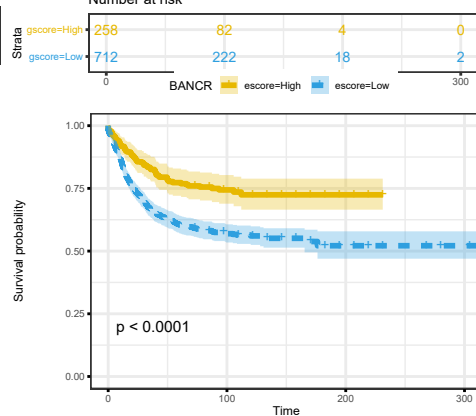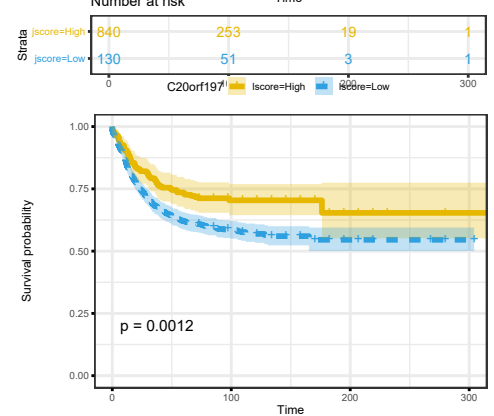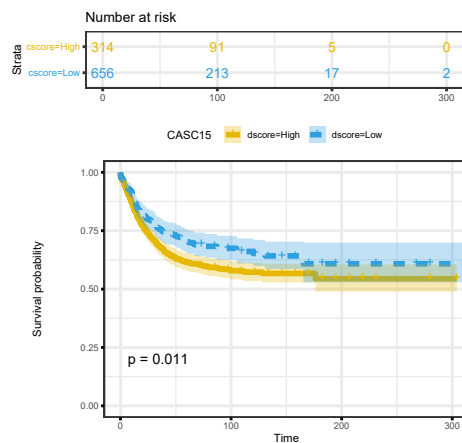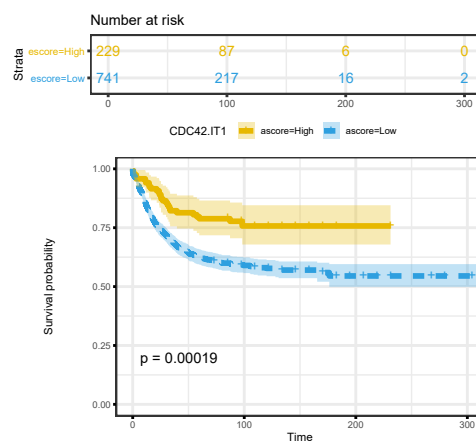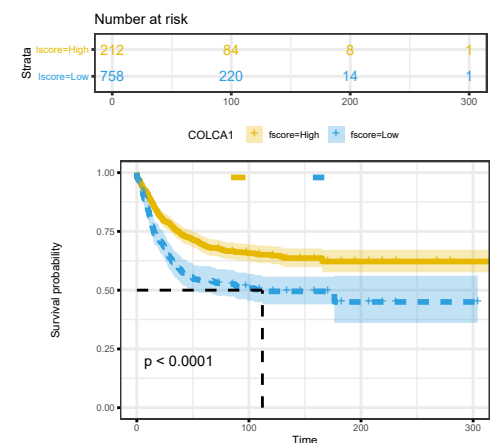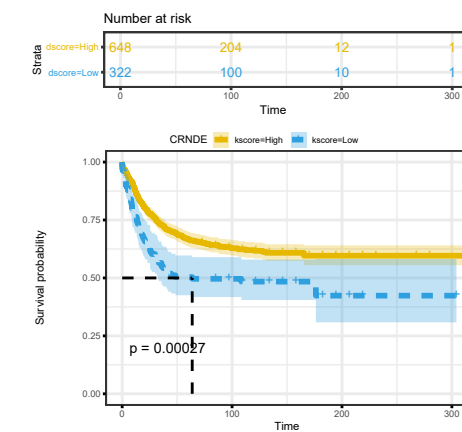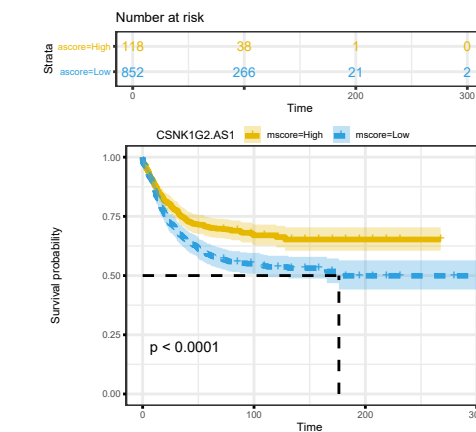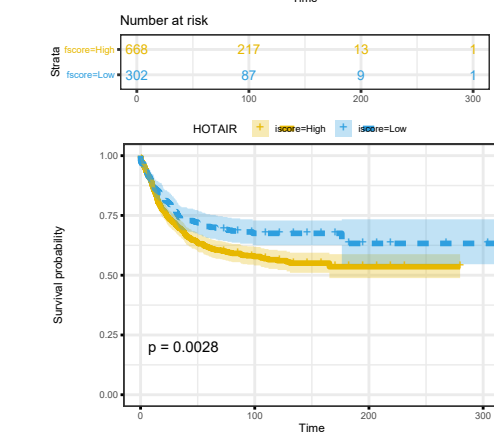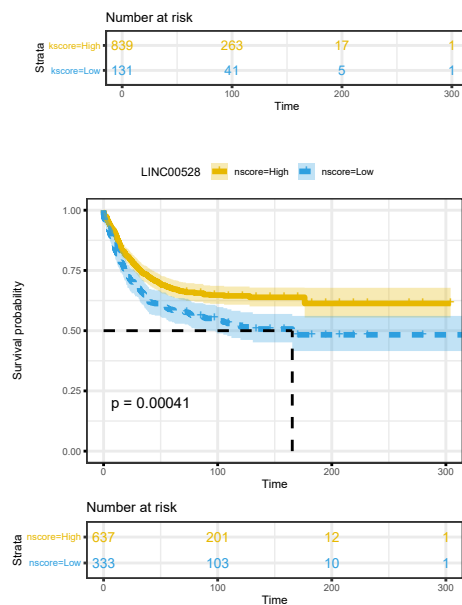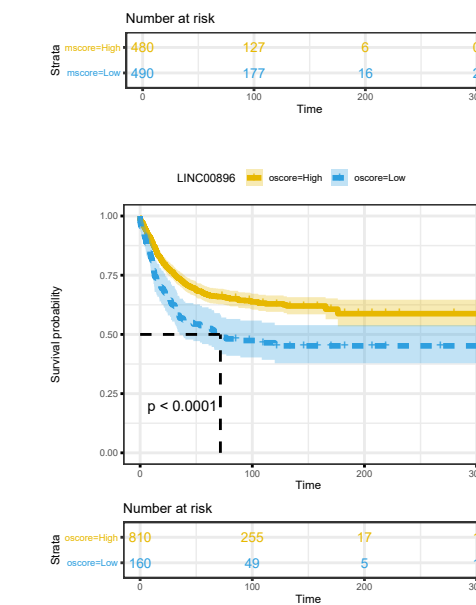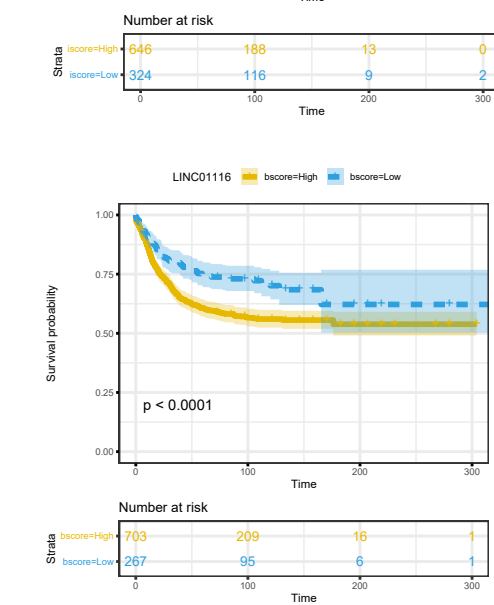

Supplement: Supplementary file 1 [file Image_1.pdf]

# Time-dependent ROC curve For OS

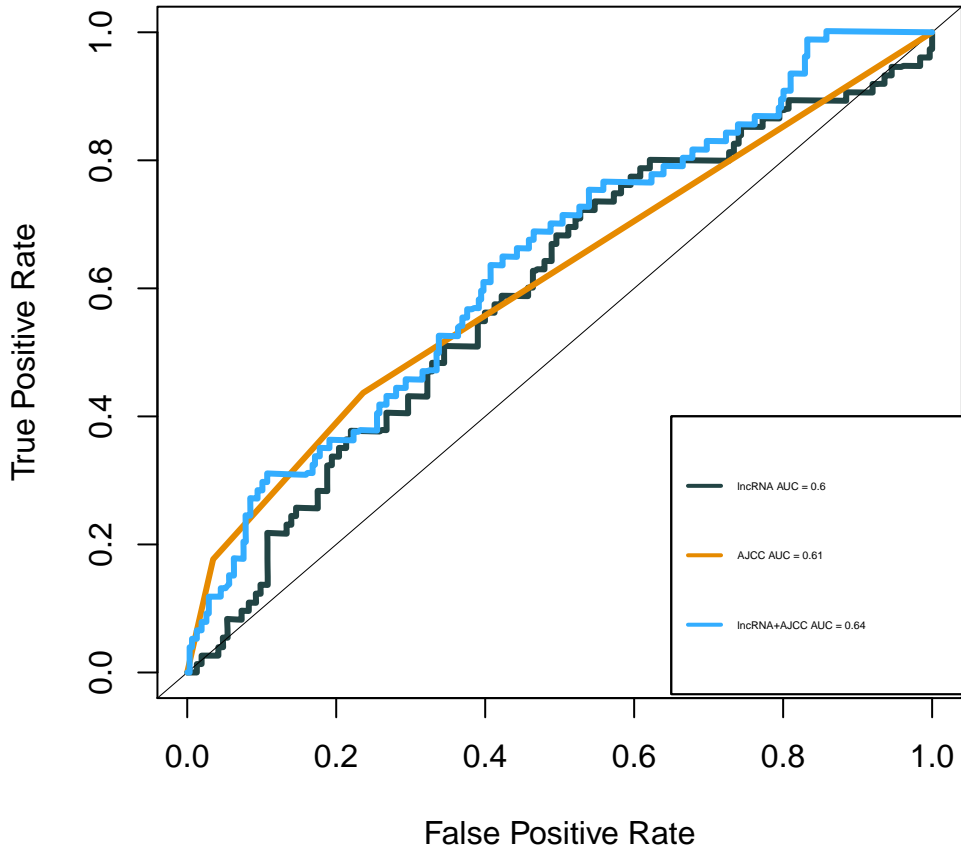

Supplement: Supplementary file 2 [file Image_2.pdf]

Group 0 1

Infiltration Abundance

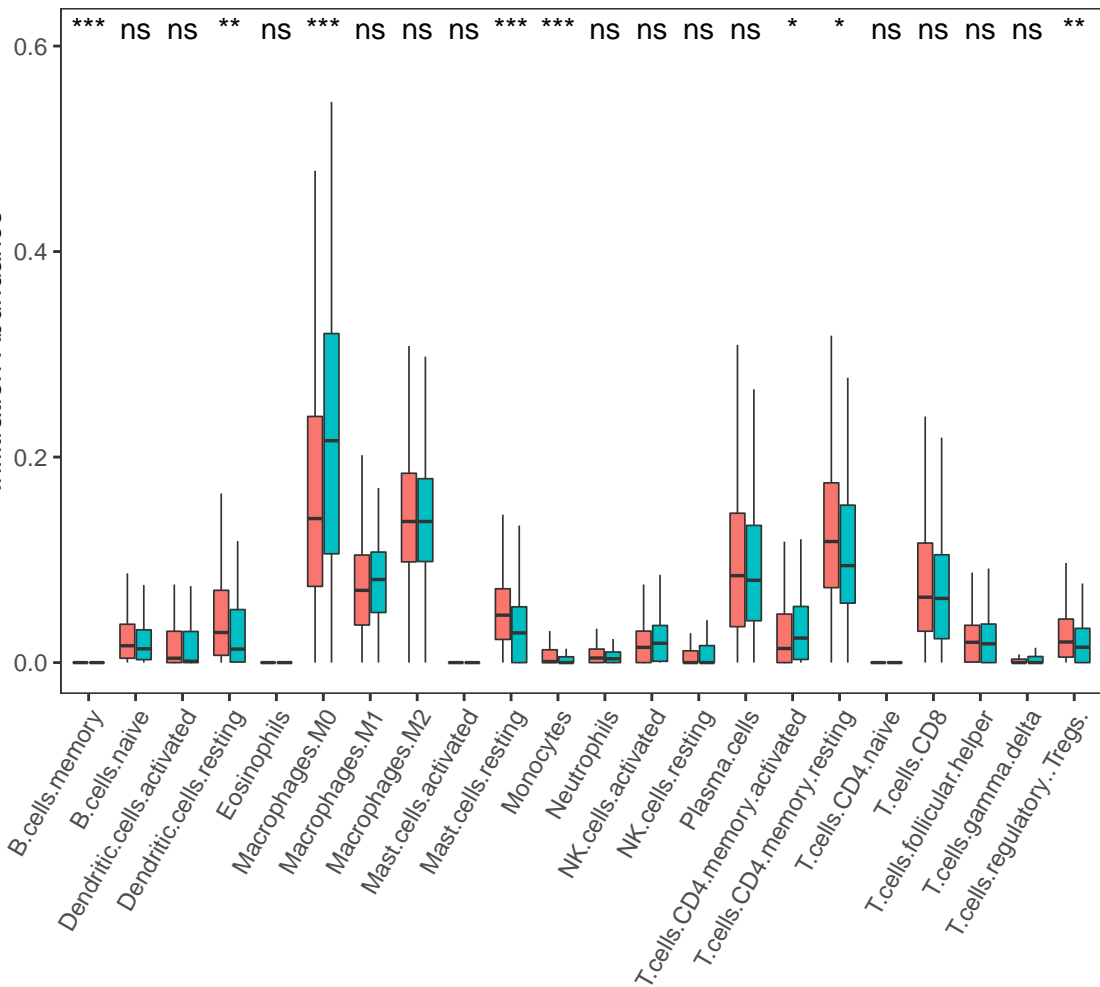

Supplement: Supplementary file 3 [file Image_3.pdf]

A  
Altered in 56 (98.25%) of 57 samples.

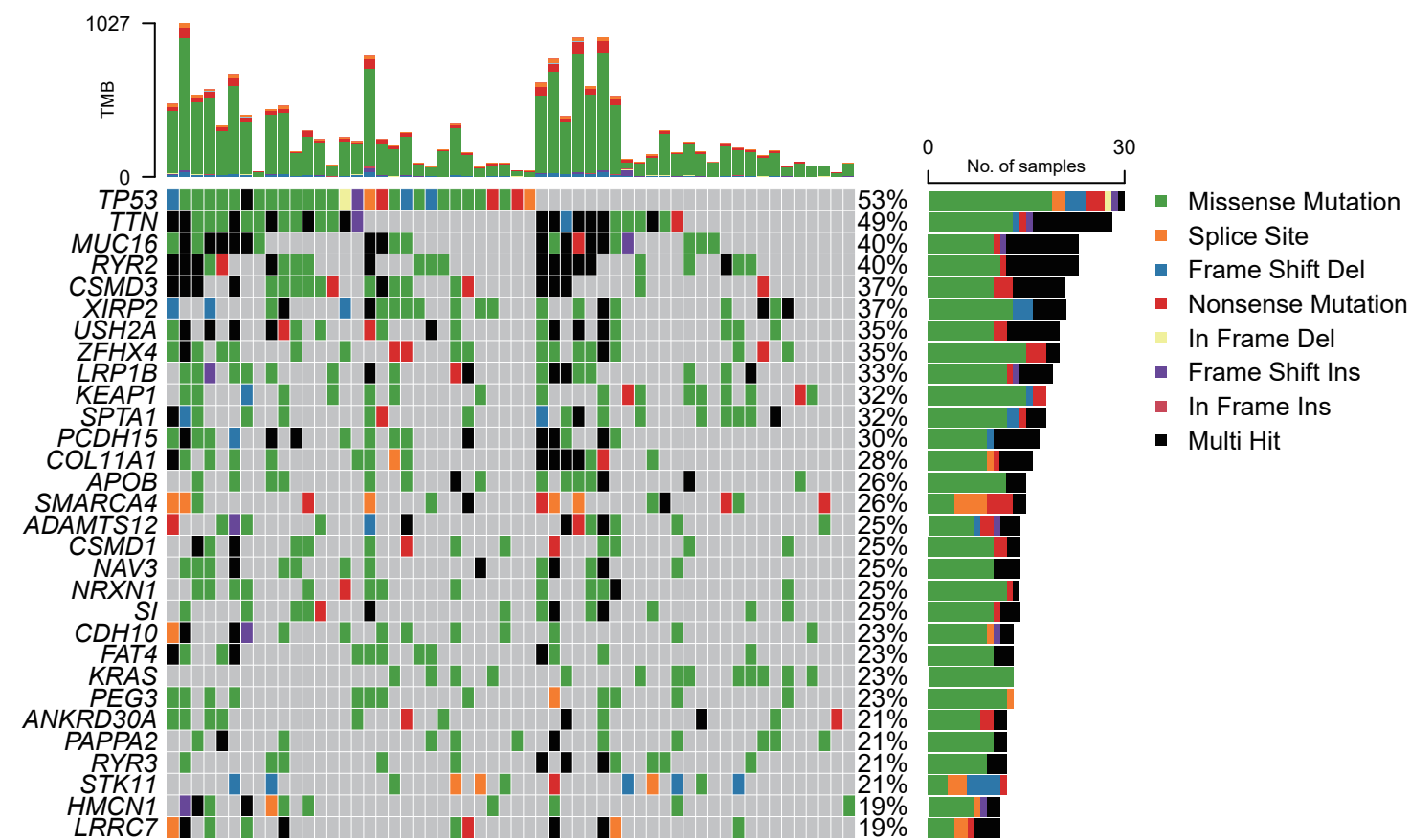

B  
Altered in 382 (92.49%) of 413 samples.

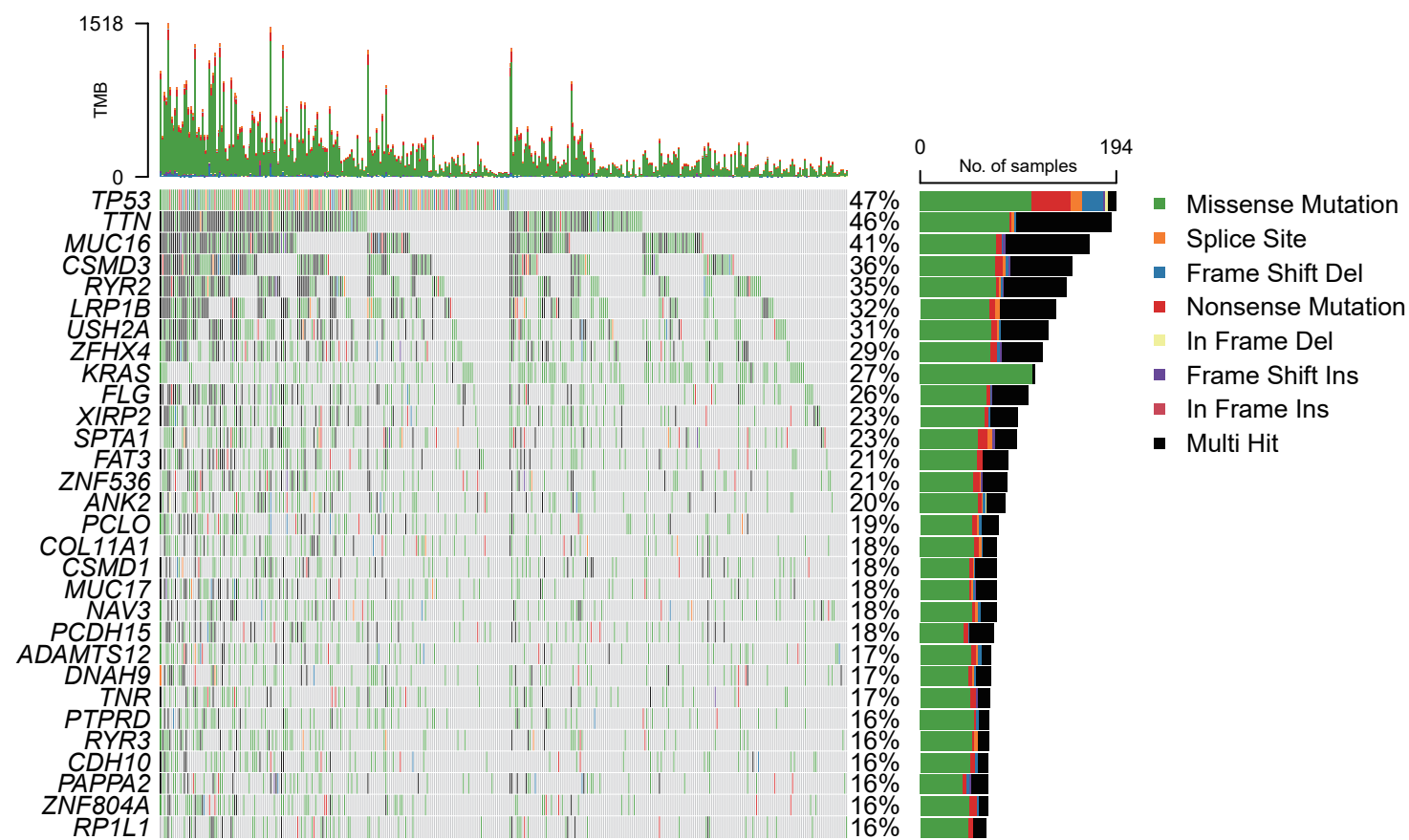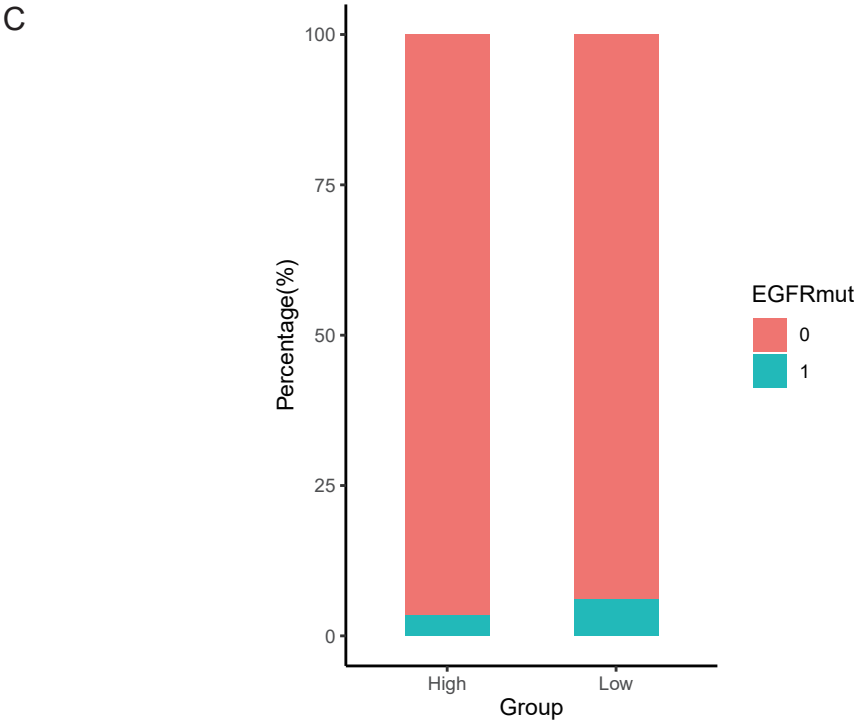

Supplement: Supplementary file 4 [file Image_4.pdf]

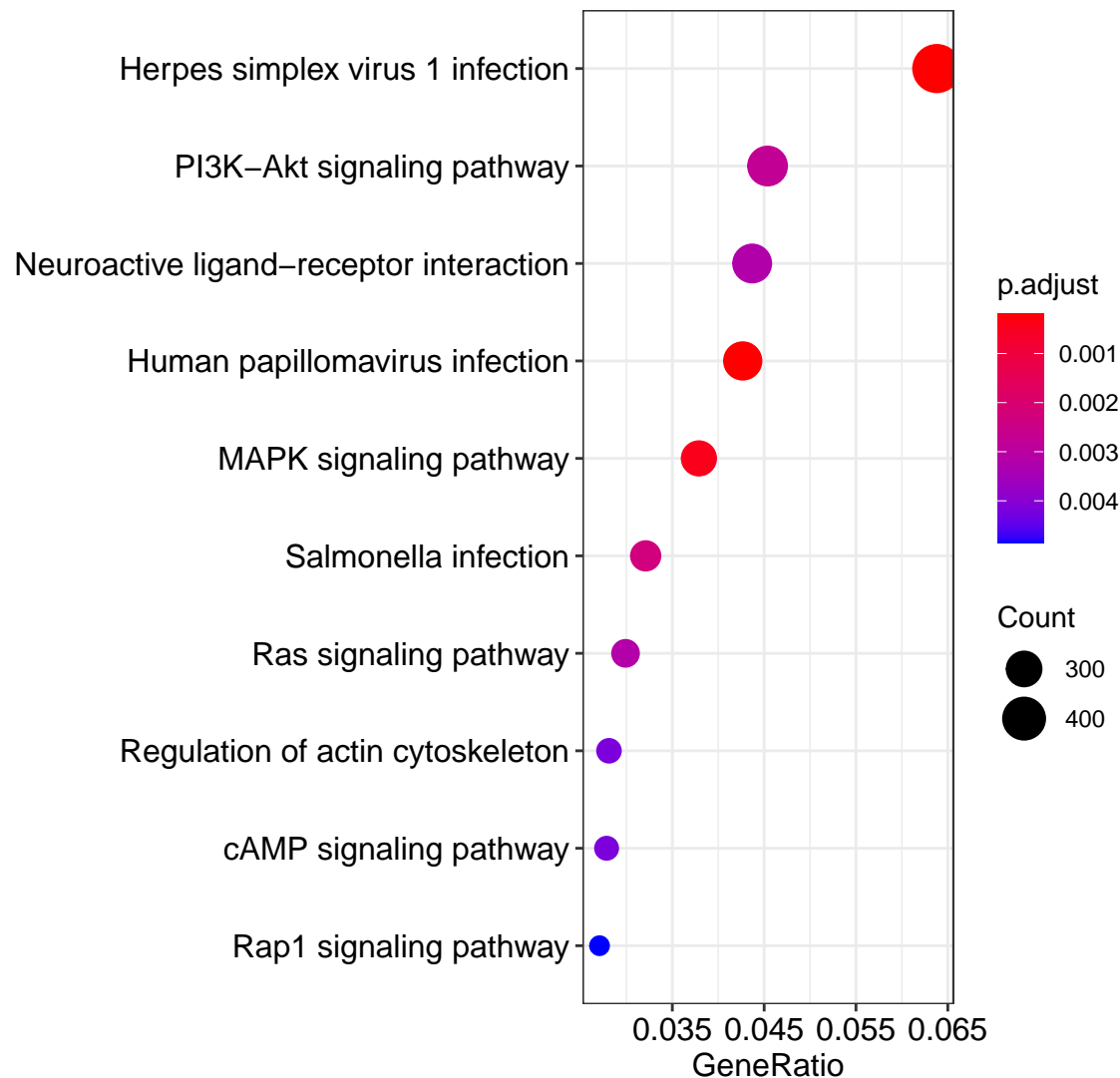

Supplement: Supplementary file 5 [file Image_5.pdf]

**A**

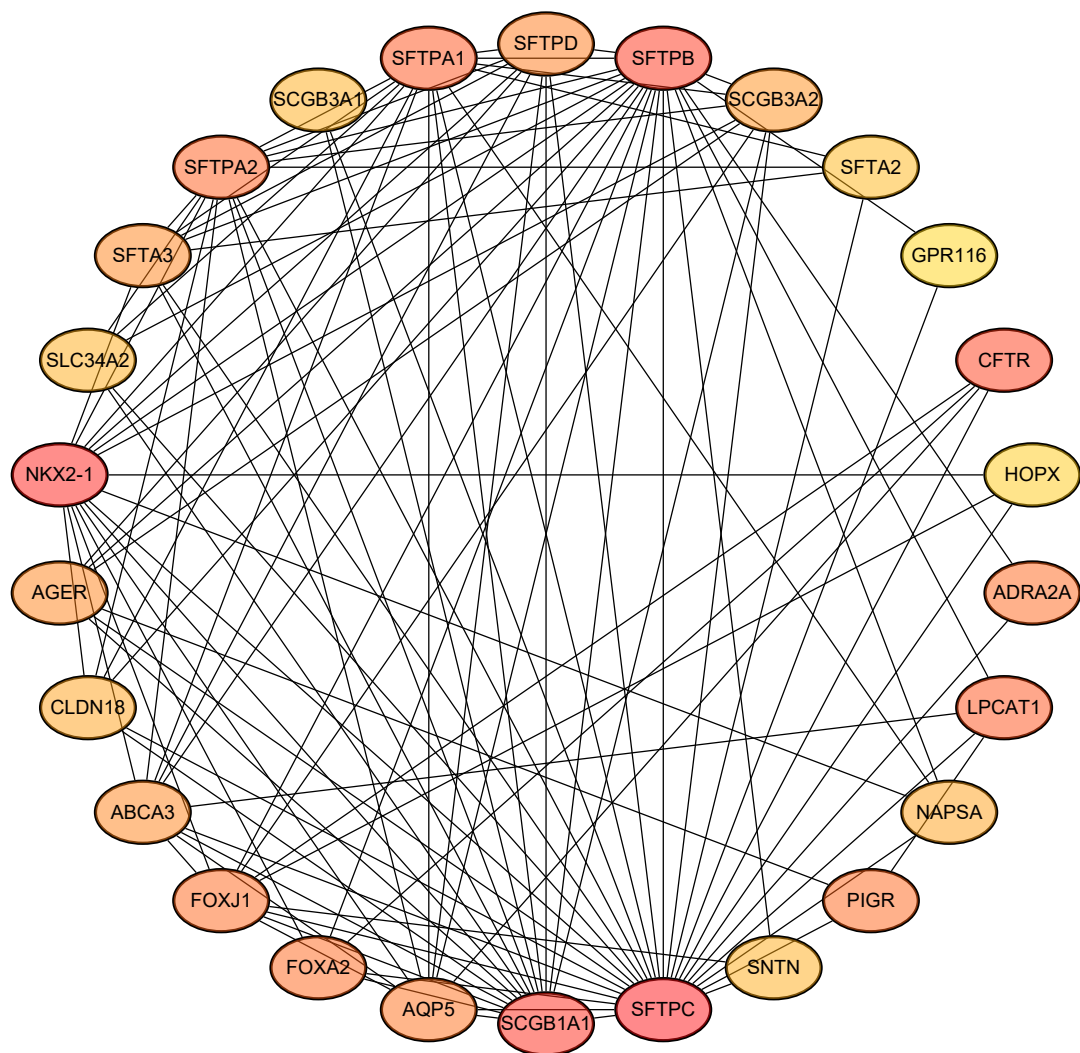

**B**

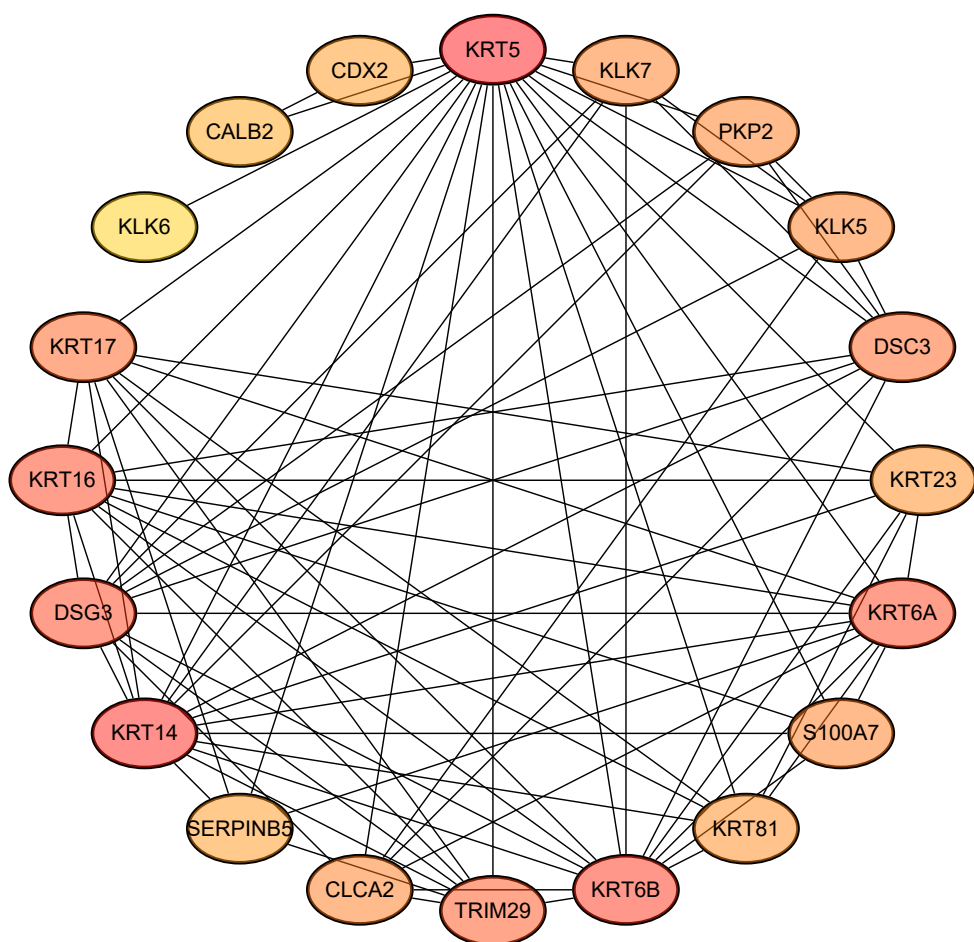

Supplement: Supplementary file 6 [file Image_6.pdf]

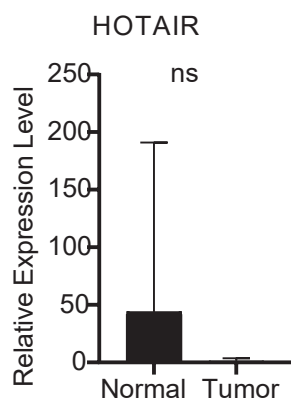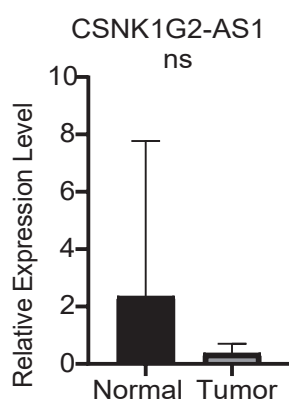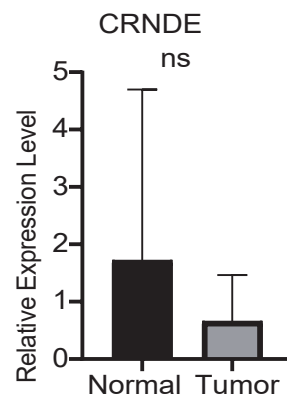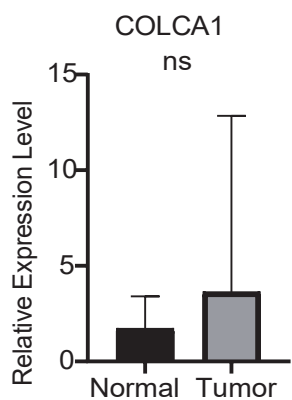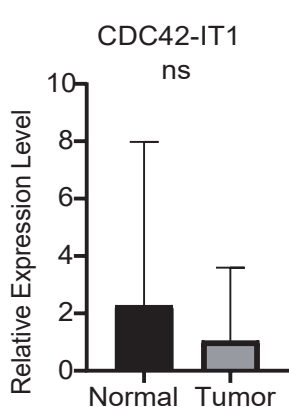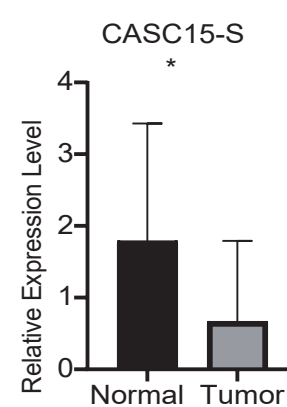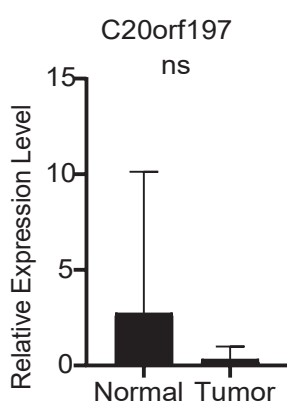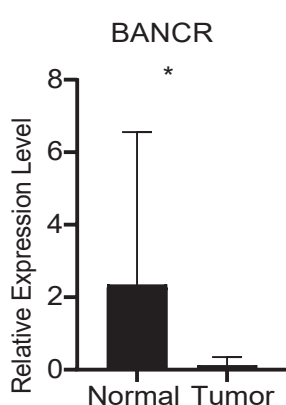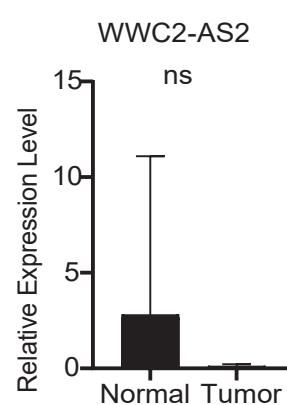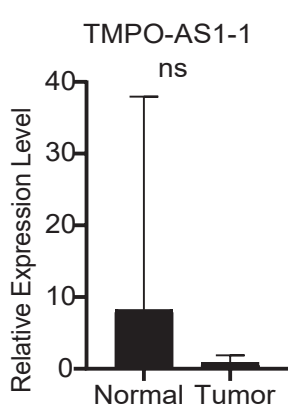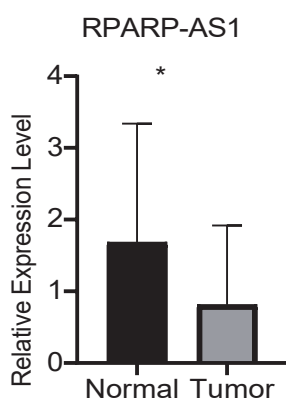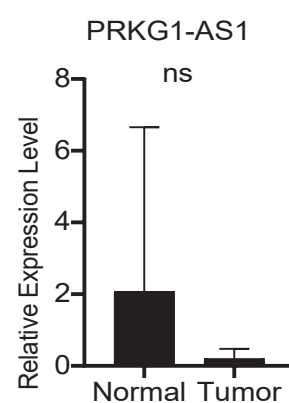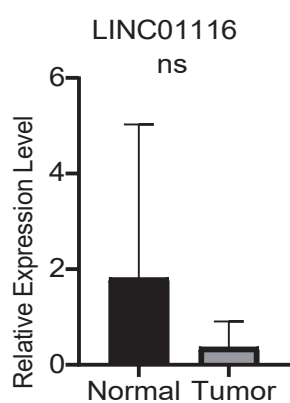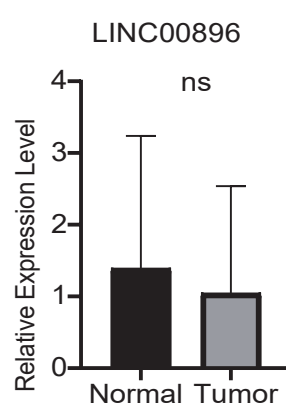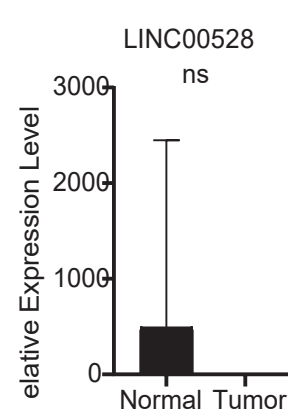

Supplement: Supplementary file 7 [file Image_7.pdf]
